# Al-doped ZnO/Ag-nanowire Composite Electrodes for Flexible 3-Dimensional Nanowire Solar Cells

**DOI:** 10.1038/s41598-017-07180-1

**Published:** 2017-08-21

**Authors:** Minoli K. Pathirane, Hadi Hosseinzadeh Khaligh, Irene A. Goldthorpe, William S. Wong

**Affiliations:** 1 GLOBALFOUNDRIES Inc., Colleges of Nanoscale Science and Engineering, 257 Fuller Road, Albany, New York, USA 12203; 2 Department of Electrical and Computer Engineering, University of Waterloo, 200 University Avenue West, Waterloo, Ontario, Canada N2L3G1

## Abstract

Silver nanowires in conjunction with sputter-coated Al-doped ZnO (AZO) thin films were used as a composite transparent top electrode for hybrid radial-junction ZnO nanowire/a-Si:H p-i-n thin-film solar cells. Solar cells with the composite nanowire top contacts attained a short-circuit current density (J_sc_) of 13.9 mA/cm^2^ and a fill factor (FF) of 62% on glass substrates while a J_sc_ of 13.0 mA/cm^2^ and FF of 62% was achieved on plastic substrates. The power conversion efficiency (PCE) of the 3-dimensional solar cells improved by up to 60% compared to using AZO electrodes alone due to enhanced coverage of the top electrode over the 3-D structures, decreasing the series resistance of the device by 5×. The composite layer also showed a 10× reduction in sheet resistance compared to the AZO thin-film contact under applied mechanical strain.

## Introduction

Three-dimensional (3-D) radial-junction nanowire (NW) solar cells (NWSCs) allow for efficient charge extraction of minority carriers along the radial direction of the nanowire while offering increased optical absorption along its length. The effectiveness of this geometry has been demonstrated with crystalline silicon (c-Si)^1–4^, hydrogenated amorphous silicon (a-Si:H)^5–8^, and hybrid ZnO nanowire/a-Si:H thin-film nanowire arrays^1, 9–12^. However, the 3-D structure makes it difficult to attain a conformal transparent top contact on the solar cell device using conventional physical-vapor deposition (PVD) techniques such as sputtering^9^. Shadowing effects by neighboring nanowires often result in a discontinuous electrical top contact to the devices, resulting in high series resistance and poor charge collection.

Silver NWs have been employed as an alternate electrode in planar organic^13–15^ and inorganic^16, 17^ devices. Their application in 3-D nanowire radial junction device structures however, has been limited. The flexibility of the Ag NWs has been exploited to form conformal top transparent contacts for 3-D ordered Si NWSC arrays^18^. The Ag nanowires mechanically sag within the gap between NWSCs to physically wrap themselves around the radial-junction solar cell. While this technique provided good electrical contact to the NWSC, we found that this approach is not suitable for high density or disordered nanowire arrays; the close proximity of adjacent radial-junction devices prevents the Ag NWs from completely wrapping around the device, resulting in poor electrical contact. In this paper, a composite electrode consisting of Ag NWs and sputter-coated Al-doped ZnO (AZO) was used to create composite top electrodes on high density, disordered hybrid radial-junction NWSCs on flexible substrates. The nanowire device consisted of a ZnO nanowire core that was coated with an a-Si:H p-i-n thin-film layer to create a 3-D thin-film solar cell. The ZnO core protrudes out of the plane of the substrate surface to create a 3-D network that was used to enhance light scattering within the a-Si:H p-i-n shell coating^9, 19^. The conformal composite electrode was created through a low-temperature annealing process (200 °C) to soften and sinter the Ag NWs on the solar cell device. The composite electrode had >85% optical transparency and up to 5× lower series resistance compared to AZO thin-film electrodes. Furthermore, the mechanical flexibility of the Ag NWs enabled a 10× lower sheet resistance in the composite film compared to the AZO when the films were mechanically bent, permitting flexible 3-D NWSCs.

## Experimental

**Figure 1:**
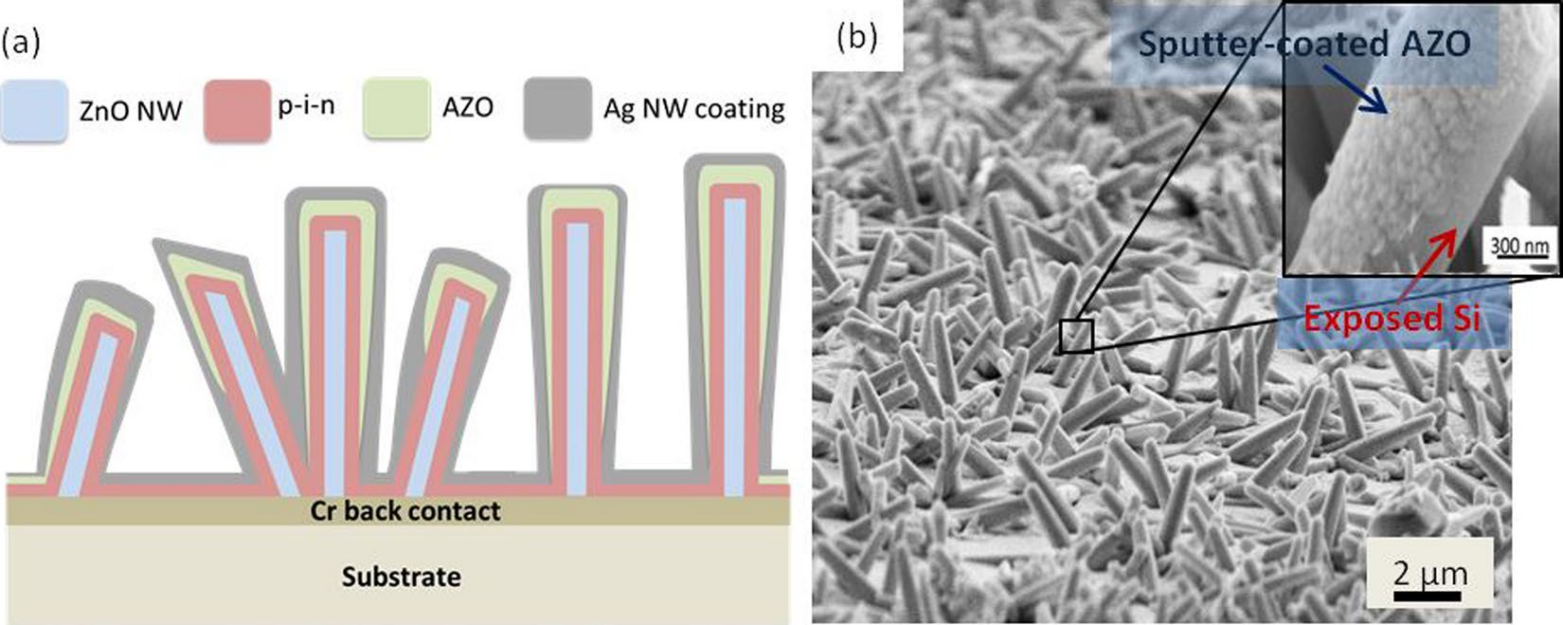
(**a**) Schematic diagram of the 3-D NWSCs with 40 nm p+, 300 nm i, and 40 nm n+ a-Si:H coated on 2 µm long ZnO NWs, which were grown on 100 nm sputter-coated Cr. (**b**) SEM image of the NWSCs before Ag NW coating. Inset: SEM image depicting poor AZO film coverage on the NWSC sidewall.

Substrate-oriented hybrid NWSCs were fabricated in a two-step process by first growing disordered ZnO NWs in a hydrothermal bath followed by vacuum deposition of a-Si:H p-i-n thin-film photodiodes; details of the fabrication process are provided elsewhere^9^. Briefly, the disordered out-of-plane 2 µm long ZnO nanowire mats grown on both glass and polyethylene naphthalate (PEN) substrates, which were sputter-coated with 100 nm of Cr, were lithographically patterned and coated with an a-Si:H p-i-n thin film (40 nm p^+^, 300 nm i, and 40 nm n^+^ a-Si:H) by plasma-enhanced chemical-vapor deposition (PECVD) to form an a-Si:H radial-junction photodiode. An 80 nm thick AZO thin film was sputtered on the NW devices as the top transparent electrode. Following the AZO deposition, Ag NWs (Blue Nano Inc. ~10 µm long, 35 nm in diameter) dispersed in ethanol (4 mg/mL concentration), were coated onto the AZO electrode using the Mayer rod technique^20–22^. The thickness of the Ag NW layer was varied by controlling the number of NW coatings onto the AZO surface. The device structure was subsequently annealed for 1 hour in a N_2_ chamber between 120 °C to 240 °C to create a thin-film coating on the solar-cell devices. A reactive ion etch was performed to expose the back Cr electrode and define the device active area. A cross-sectional schematic of the 3-D solar cell structure is illustrated in Fig. 1a. NWSCs with an 80 nm thick sputter-coated AZO top electrode without Ag NWs were used as reference cells. Planar cells were also fabricated having the same a-Si:H thin-film p-i-n layers used in the hybrid NWSC structures. In these devices, the a-Si:H p-i-n layers were deposited directly onto the Cr bottom contact and the top transparent electrode was an Al-doped ZnO layer.

The Ag NWs and the devices were probed using a Keithley 4200-SCS parameter analyzer. Four-point probe and profilometry measurements were used to determine the conductivity and thickness of the AZO thin films and composite films, respectively. The electrical measurements of the nanowire devices on the PEN substrates were performed under mechanical strain by attaching the structures onto curved static test fixtures having a radius of curvature, *R*, of 38 mm, 32 mm, 16 mm, 6 mm, and 4 mm. Multiple data points were measured at different locations of the samples to provide statistical data for the electrical measurements; the mean values of these measurements are reported.

The morphology, orientation, and density of the NWSC structures and their contacts were investigated using a LEO 4400 field-emission scanning electron microscope (FE-SEM) and a Reichert Polylite 88 optical microscope. Plan-view SEM micrographs were converted into high-contrast digital images (bright regions represented the nanowires and dark regions for the background) to determine the NW density from the ratio of bright-to-dark pixels, using the imaging software, ImageJ. Optical properties of the film coatings and device structures were obtained using an integrated sphere with a photomultiplier tube and PbS InGaAs detectors. Optical and electrical characteristics of the AZO thin-film and composite AZO/Ag NW layer were performed on planar test structures without an active device layer. Electrical I-V characteristics of the fabricated NWSCs and planar devices were extracted using an AM 1.5 G illumination solar simulator (ABET Technologies 2000) and the external quantum efficiency (EQE) was measured using a monochromatic light source (PV Measurements) calibrated with a reference Si solar cell.

## Results and Discussion

Figure 1b depicts a SEM micrograph of the NWSC array sputter-coated with the AZO top electrode; the inset is a magnified view of a sidewall of a NWSC, revealing the discontinuous nature of the thin-film contact (the spotted high-contrast regions along the NW sidewall) due to the PVD processing. Before applying the Ag NWs onto the NWSC devices, the dependence of the optical transmittance of the Ag NW layer and its electrical conductivity on the number of Ag NW coatings were investigated.

The average optical transmission in the visible spectrum of a transparent electrode can be represented as,

1 $$T({\lambda }_{av})={(1+\frac{188.5}{{R}_{sheet}}\frac{{\sigma }_{op}({\lambda }_{av})}{{\sigma }_{DC}})}^{-2},$$

where the sheet resistance, R_sheet_ = *ρ*/*t*, with *t* being the thickness of the layer and *ρ* being the resistivity of the transparent thin film^16, 23^. The ratio between the DC electrical and optical conductivity, *σ*_*DC*_/*σ*_*op*_, may be used to directly compare different coatings fabricated using multiple materials and different processing conditions^24^. A high *σ*_*DC*_/*σ*_*op*_ corresponds to a condition where the incoming light is minimally obstructed before reaching the underlying layer while also possessing a low R_sheet_.

**Figure 2:**
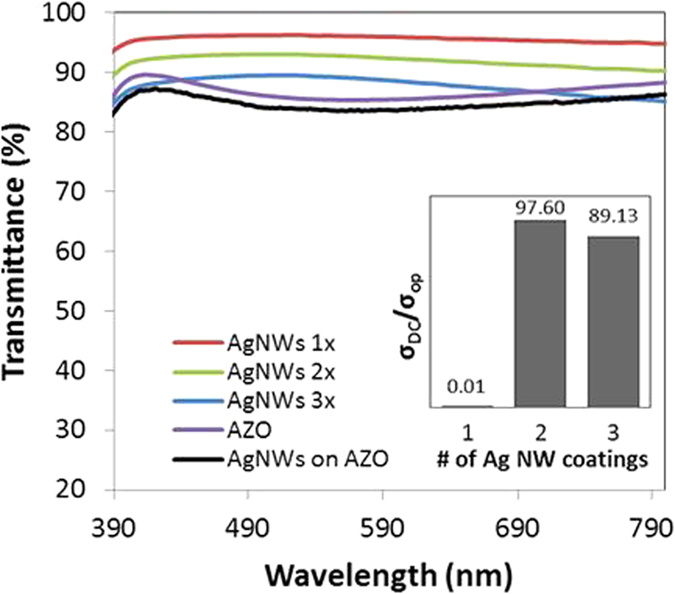
A comparison of the transmittance spectra of Ag NWs with various coating layers, a sputter-coated AZO thin-film, and the composite AZO/Ag NW layer with Ag NWs coated twice. Inset: A plot of the figure of merit (σ_DC_/σ_op_) vs. the number of Ag NW layers.

The optical transmittance at wavelengths from 390 nm to 800 nm for layers consisting of: (1) multiple coatings of Ag NWs, (2) an 80 nm thick AZO film, and (3) an AZO/Ag NW composite layer on a planar surface are shown in Fig. 2. The *σ*_*DC*_/*σ*_*op*_ for each of the Ag NW coatings was used to determine relative changes in the Ag NW percolation network as a function of the number of coating layers (Fig. 2). The *σ*_*DC*_/*σ*_*op*_ achieved for 1 layer of Ag NWs (density ~2 × 10^7^ NW/cm^2^) was determined to be below the percolation threshold due to a low effective thickness, resulting in a high Rsheet (560 kΩ/sq)^25^ A maximum *σ*_*DC*_/*σ*_*op*_ was obtained from a dual coating of Ag NWs (density ~4.3 × 10^7^ NW/cm^2^). Beyond two layers, the *σ*_*DC*_/*σ*_*op*_ decreased, suggesting that a relative percolation network having a Ag NW density near 4.3 × 10^7^ NW/cm^2^ is optimum.

Ag NWs were coated on the NWSC structures in anticipation that the mechanical flexibility of the Ag NWs would allow conformal coverage over the solar cells. On the contrary, SEM characterization of the Ag NW coating onto the NWSC sidewalls revealed that the Ag NWs did not form a conformal coating but rather they were observed to contact only the tips of the NWSC structures. This result was dramatically different compared to the report by Turner *et al*.^18^ due in part to the high density and disordered nature of the hybrid thin-film/nanowire solar-cell structures. The radial-junction device structures consist of a smaller pitch and higher density, which do not allow the Ag NWs to mechanically sag and wrap around the NWSCs.

**Figure 3:**
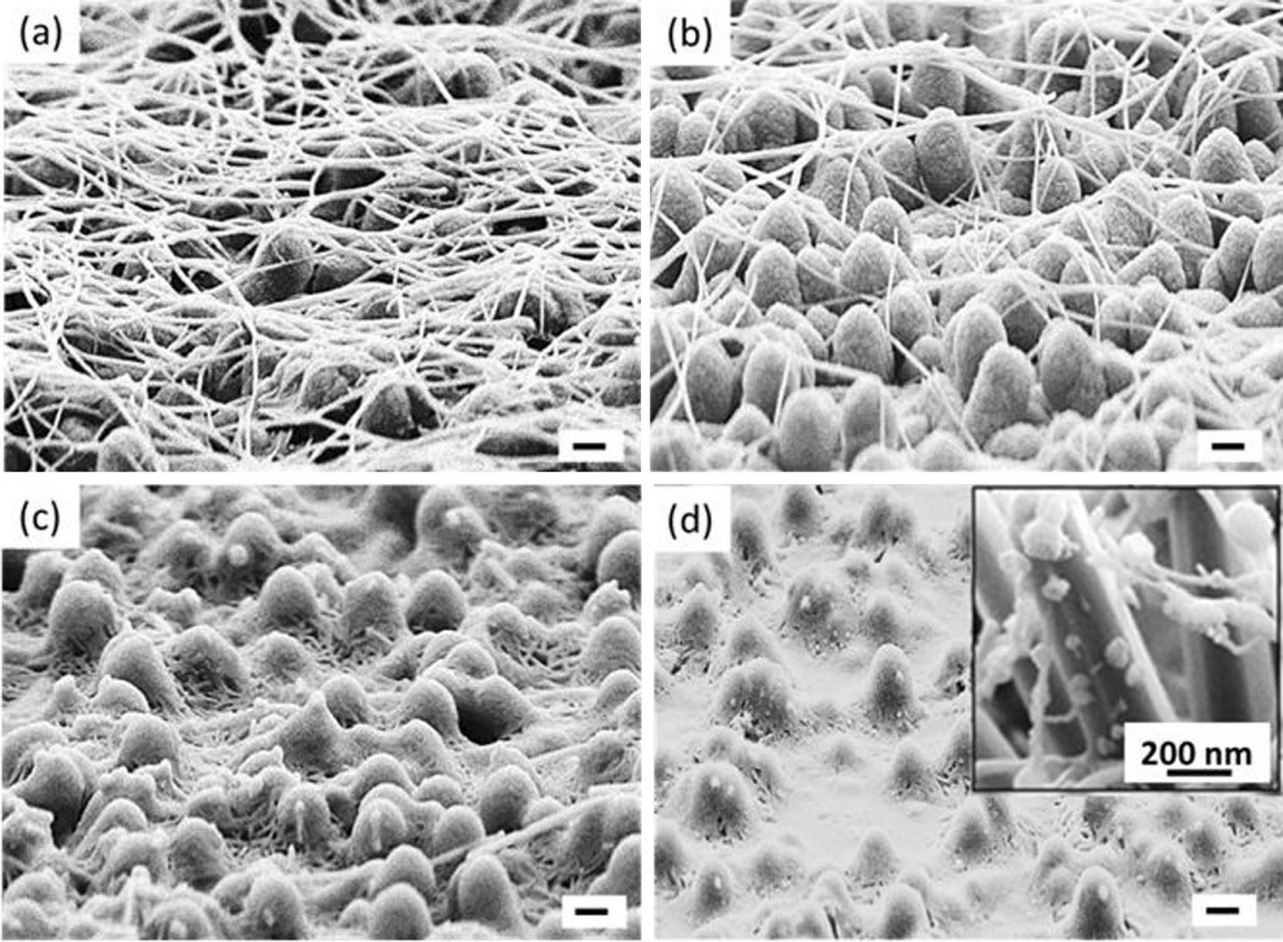
SEM images of Ag NWs coated on 3-D hybrid NWSCs using the same Ag NW coating density (2×) at annealing temperatures of (**a**) 120 °C, (**b**) 140 °C, (**c**) 200 °C, and (**d**) 240 °C. Inset of (**d**) shows a magnified SEM image of disconnected droplets of Ag on the sidewall of a 3-D nanowire after the anneal. The scale bar is 1 µm unless otherwise labelled.

To alleviate this coverage problem, the effect of annealing the coated structures was investigated. Given the relatively low sintering temperature of the Ag NWs (~190 °C), a low-temperature anneal is expected to soften the Ag NWs, allowing the nanowires to mechanically conform to a non-planar structure with a high-aspect ratio. SEM micrographs in Fig. 3 depict the effect of different annealing temperatures on the morphological changes of the Ag NWs over the 3-D structures. At 120 °C, the morphology did not change (Fig. 3a); the Ag NWs are similar to the as-coated structures that touch only the tips of the NWSC structures. By increasing the temperature to 140 °C, the Ag NWs began to soften and were observed to droop, coming into contact with the sidewalls and top surfaces of the NWSC structures (Fig. 3b). At a temperature slightly above the 190 °C sintering threshold (~200 °C), the Ag NWs were found to transform from a nanowire structure to a conformal thin-film coating over the NWSC, suggesting the sintering process allows the Ag to wet the 3-D device structure. Increasing the temperature to 240 °C resulted in melting of the Ag NWs and evidence of the Ag dewetting from the surface was observed in the form of small island clusters along the NWSC surface (inset of Fig. 3d).

**Figure 4:**
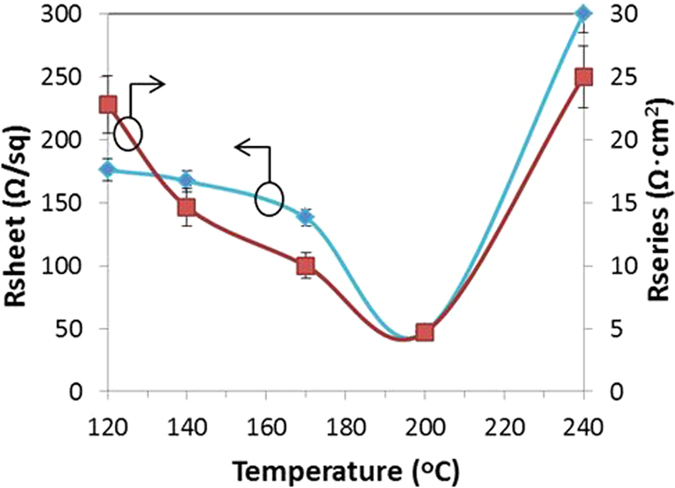
Sheet resistance of the annealed composite AZO/Ag NWs layer and series resistance values of the corresponding 3-D NWSCs with the AZO/Ag NW composite top electrode annealed at different temperatures. Note the solid lines are only guides to the eye to help separate the experimentally measured R_sheet_ and R_series_ data.

The morphological changes due to annealing also resulted in measurable electrical changes. The R_sheet_ of the AZO/Ag NW composite film and the corresponding series resistance (R_series_) of the 3-D NWSC devices as a function of the annealing temperatures are plotted in Fig. 4. Ag NW coatings that were not annealed possessed a high R_sheet_ of  >1 kOhm/sq. R_sheet_ decreased as a function of increasing temperature with a minimum of 48 Ω/sq. at 200 °C, correlating to the improved electrical contact between the nanowires and the AZO film to the underlying NW device. The drop in R_sheet_ as the temperature increases is due to the Ag NW wetting the underlying surface, forming a continuous and improved contact across the NWSC array (Fig. 3c), as well as reducing the junction resistances of the overlapping Ag NWs. As the annealing temperature is increased up to 240 °C, the Ag layer begins to dewet from the surface resulting in an increase in R_sheet_ (inset of Fig. 3d).

Additional evidence of the changes in the contact electrode with annealing temperature was observed in the measurement of R_series_ (=*V*/*I* near the open-circuit voltage (V_OC_) of the I-V characteristics under one-sun illumination). A high R_series_ of 22.8 ± 2.5 Ω·cm^2^ was measured after annealing the devices at 120 °C. However, as the anneal temperature increases (140 °C to 170 °C), R_series_ was found to decrease from 14.6 ± 2 Ω·cm^2^ to 10 ± 1.2 Ω·cm^2^, similar to the trend observed in R_sheet_. A R_series_ of 8.6 ± 2 Ω.cm^2^ was achieved at 200 °C corresponding to the wetting of the NWSC sidewalls by the Ag coating (Fig. 3c). At 240 °C, the R_series_ increased to 25 ± 3.5 Ω·cm^2^ similar to the observed increase in R_sheet_, due to the formation of island clusters (Fig. 3d).

**Figure 5:**
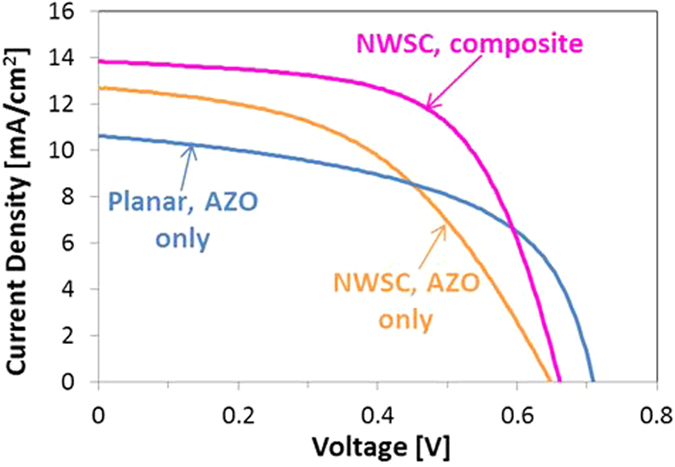
I-V characteristics obtained from (**a**) planar cells, (**b**) NWSC devices with only AZO top contacts, and (**c**) NWSC device with AZO/Ag NW composite top contacts. All devices were fabricated on glass substrates.

The extracted PCE for the NWSCs with the composite top electrode was 5.7 ± 1.2%, an enhancement of ~60% compared to the AZO-only contacts (PCE = 3.9 ± 1%). This improvement was due to several factors. First, the composite electrode has a reduced R_series_ and an increased shunt resistance (R_shunt_) (Table 1). Secondly, the increased conformal coverage of the electrodes by the Ag NWs improved charge collection resulting in higher J_SC_ and FF values (Fig. 5). Furthermore, the increased light scattering between disorderd NWSCs enhanced absorption and increased J_SC_ compared to the planar structure.

**Table 1:** Device characteristics of the NWSC with and without the AZO/Ag NW composite top electrode. Planar device characteristics are also shown for comparison.

	J_SC_ [mA/cm^2^]	V_OC_ [mV]	FF [%]	R_series_ [Ω.cm^2^]	R_shunt_ [Ω.cm^2^]	PCE [%]
Planar – AZO only	10.6 ± 1	708	54 ± 1.1	9.2 ± 1.1	386 ± 0.5	4.1 ± 1
NWSC – AZO only	12.7 ± 2	653	47 ± 1.3	18.4 ± 4.5	347 ± 10	3.9 ± 1
NWSC - composite	13.9 ± 1.5	659	62 ± 1	8.6 ± 2	813 ± 5	5.7 ± 1.2

**Figure 6:**
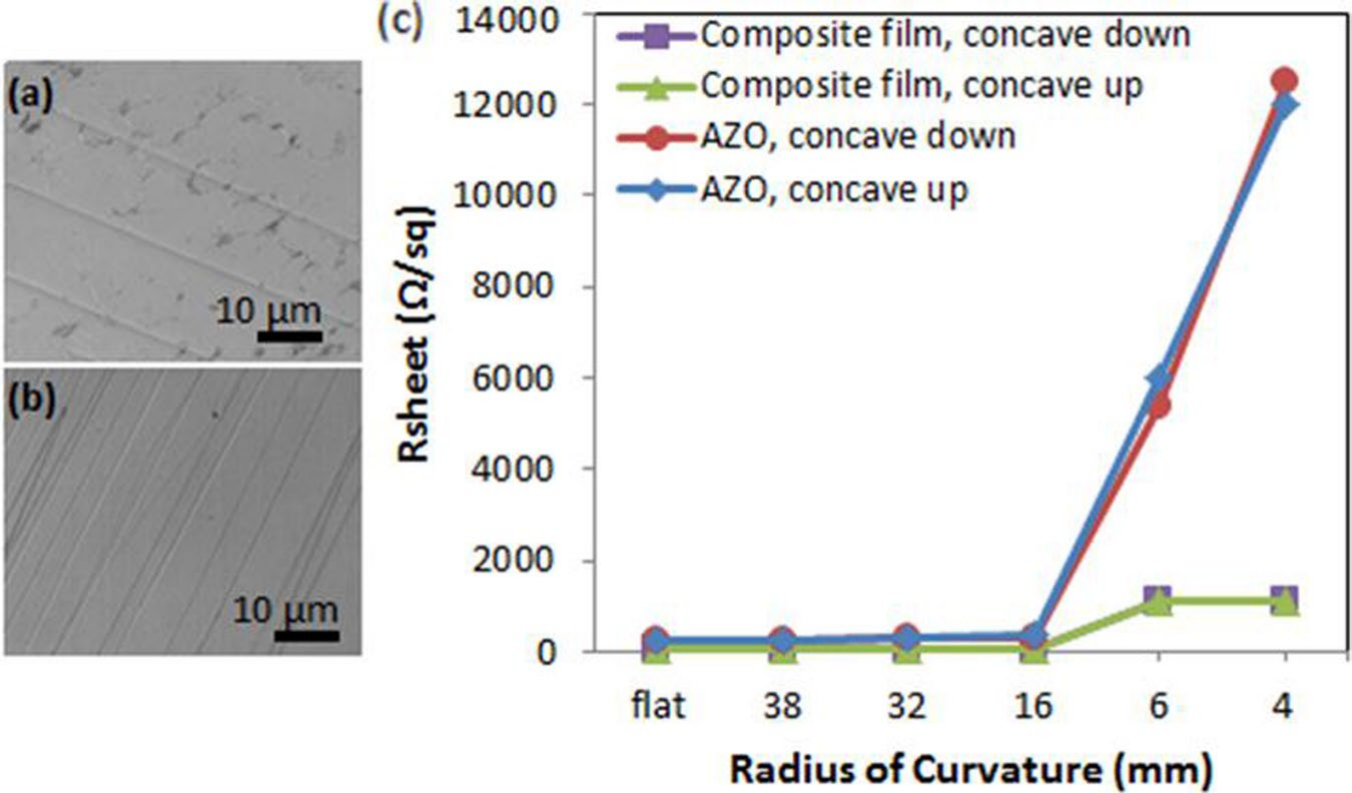
SEM micrographs at R = 6 mm of (**a**) the Ag NW/AZO composite contact and (**b**) the AZO film under tensile strain. (**c**) R_sheet_ values as a function of tensile and compressive strain states.

The AZO/Ag NW composite layer alone, without the NWSC, was also assessed on flexible polyethylene napthalate (PEN) substrates to determine the effect of mechanical strain on the electrical properties of the contact. R_sheet_ of the different electrodes was measured (Fig. 6) under bending of the PEN to various radii of curvature, *R*, from 38 mm to 4 mm in both concave-up (*i*.*e*. bending of the flexible substrate upwards to the incoming light) and concave-down (*i*.*e*. bending downward from the incoming light) orientations to produce compressive and tensile strain states in the film, respectively. SEM micrographs of the planar AZO thin-film under tension exhibited cracking at a radius of curvature, *R* = 32 mm. On the other hand, the sintered composite AZO/AgNW electrodes did not show cracking until *R* = 6 mm, with a calculated strain of 2%. A change in R_sheet_ by ~2.5% was observed when the film was bent to *R* = 38 mm and *R* = 32 mm for both contact layers. Comparing the two contact materials, the AZO-only electrode failed under a strain energy of 0.2 mJ/m^3^ while the composite layer failed at a strain energy of 5.9 MJ/m^3^.

In terms of the electrical properties of the planar contacts, R_sheet_ = 1.1 ± 0.2 kΩ/sq for the AZO/AgNW composite contact at *R* = 4 mm, while a significantly larger R_sheet_ (12.5 ± 2 kΩ/sq) was measured for the AZO-only contacts at this bending radius. The ~10× lower R_sheet_ for the composite layer is likely due to the increased ductility of the Ag NW contact that allows for a more robust contact compared to the AZO-only electrodes under mechanical strain.

**Figure 7:**
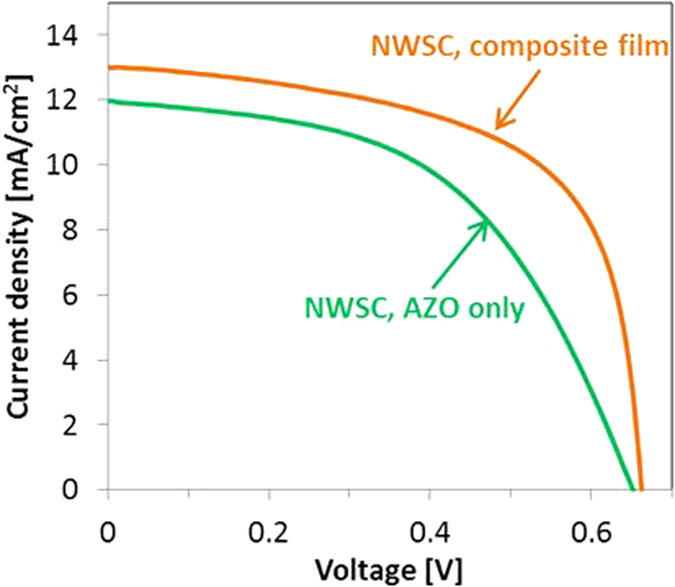
I-V characteristics of flexible 3-D NWSCs with an AZO/Ag NW composite top electrode and an AZO thin-film only as top electrodes.

As a demonstration of the effectiveness of the composite electrodes for flexible NWSC devices, 3-D NWSCs on PEN substrates were fabricated with the same low-temperature process used for the NWSCs on glass. The electrical performance from the flexible disordered NWSCs with the AZO/Ag NW composite top electrode was compared to the 3-D NWSCs fabricated with the AZO thin-film top electrode (Fig. 7). The device with the AZO/Ag NW electrode has a J_SC_ of 13.1 ± 1.1 mA/cm^2^, close to that obtained from the NWSCs on glass and a PCE of 5.5 ± 1.1% (Table 2). Similar to the device results on glass substrates, the PCE was improved by sintering the Ag NWs to reduce the contact resistance, demonstrating the feasibility of fabricating mechanically flexible 3-D NWSCs on arbitrary substrates.

**Table 2:** Electrical characteristics of flexible 3-D NWSCs with the AZO/Ag NW composite film electrode and AZO thin-film electrode.

	J_SC_ [mA/cm^2^]	V_OC_ [mV]	FF [%]	PCE [%]
NWSC–AZO only on PEN	12.0 ± 1	656	51 ± 1.5	4.0 ± 1
NWSC–composite film on PEN	13.1 ± 1.1	662	63 ± 2	5.5 ± 1.1

## Conclusions

In conclusion, a conformal and transparent AZO/AgNW top electrode was used to improve electrical contact for 3-D NW solar cells while maintaining high optical transmission. The efficacy of the Ag NW contact, both structurally and electrically, was found to be a function of the annealing temperature. An annealing temperature of 200 °C resulted in the sintering of the Ag NWs to form a continuous and conformal contact to the 3-D NWSC structures that resulted in minimizing R_sheet_ of the contact. The composite top electrode in the NWSCs fabricated on glass substrates resulted in a total PCE to 5.7 ± 1% compared to 3.9 ± 1% from the NWSCs that only used AZO thin-film electrodes. 3-D devices on PEN substrates resulted in a PCE of 5.5 ± 1.1% using the composite contact compared to 4 ± 1% with the AZO-only electrodes. The increased PCE values achieved was attributed to the improved electrical contacts provided by the composite electrode. The composite layer also showed enhanced mechanical flexibility over the AZO thin film due to the lower elastic modulus of Ag NWs compared to AZO thin film. The latter degraded with increasing mechanical strain while the composite layer showed more stable characteristics under bending for a radius of curvature down to 4 mm.

### Data availability statement

The datasets generated during and/or analysed during the current study are available from the corresponding author on reasonable request.
